# Tuberculosis and Sexual and Reproductive Health of Women in Four African Countries

**DOI:** 10.3390/ijerph192215103

**Published:** 2022-11-16

**Authors:** Rayan Korri, Abhishek Bakuli, Olumuyiwa A. Owolabi, Julieth Lalashowi, Cândido Azize, Mohammed Rassool, Farzana Sathar, Andrea Rachow, Olena Ivanova

**Affiliations:** 1 Division of Infectious Diseases and Tropical Medicine, Medical Centre of the University of Munich (LMU), 80802 Munich, Germany; 2 Medical Research Council Unit, The Gambia at the London School of Hygiene and Tropical Medicine, Banjul P.O. Box 273, The Gambia; 3 National Institute for Medical Research (NIMR), Mbeya Medical Research Centre, Mbeya P.O. Box 2410, Tanzania; 4 Instituto Nacional de Saúde (INS), Marracuene 3943, Mozambique; 5 Clinical HIV Research Unit, Department of Internal Medicine, School of Clinical Medicine, Faculty of Health Sciences, University of Witwatersrand, Johannesburg 2092, South Africa; 6 The Aurum Institute, Johannesburg 2194, South Africa; 7 German Center for Infection Research (DZIF), Partner Site Munich, 80802 Munich, Germany

**Keywords:** tuberculosis, sexual and reproductive health, pregnancy, Africa

## Abstract

Tuberculosis (TB) is a major reason of maternal mortality in low-income countries, and it increases the probability of adverse sexual and reproductive health (SRH) outcomes, including ectopic pregnancy and perinatal mortality. The data presented here is from the TB Sequel observational cohort conducted in four African countries. For this sub-study, we selected only female participants, who were diagnosed with drug susceptible TB and followed-up until the end of anti-TB treatment. The data collection included questionnaires, clinical examination and laboratory tests at TB diagnosis, day 14, month 2, 4 and 6. A total of 486 women, with 88.3% being 18–49 years old, were included in the analysis. Around 54.7% were HIV positive. Most of the participants (416/486; 85.6%) in our cohort were considered cured at month 6. Only 40.4% of non-pregnant women of reproductive age used contraception at TB diagnosis. A total of 31 out of 486 women experienced pregnancy during TB treatment. Pregnancy outcomes varied between live birth (16/31; 51.6%), induced abortion (6/31; 19.4%), miscarriage (4/31; 12.9%) and stillbirth (3/31; 9.6%). Integration and linking of SRH services with TB programmes are vital to increase contraception use and protect women from obstetric risks associated with pregnancy during TB treatment.

## 1. Introduction

Tuberculosis (TB) is considered the 13th principal cause of death and the infectious disease with the highest mortality rate [1,2]. In 2020, nearly 10 million individuals were infected with TB and 1.5 million died because of it [2,3]. Two-thirds of the total TB patients worldwide live in eight countries, Nigeria and South Africa being the ones located in the World Health Organization (WHO) African Region [1,2]. Although men are more impacted by TB compared to women, with 56% of worldwide TB cases in 2020, the elevated percentage of women of reproductive age with TB is considered alarming [2]. There is an accumulating body of evidence on TB during pregnancy and the importance of early TB diagnosis. A number of studies highlight the problem of the delayed diagnosis of TB due to “non-typical” symptoms during pregnancy [4]. Moreover, in low-income countries, TB is a major reason for maternal mortality [5,6,7] and women affected by TB have an increased probability to experience an ectopic pregnancy, premature birth and perinatal mortality [8].

TB is also the leading cause of morbidity and mortality among people living with HIV [3] and the greatest burden of both HIV and TB occurs in women of reproductive age (15–49 years) [9]. The rate of TB disease among pregnant women living with HIV is higher than that among pregnant women without HIV [10,11]. Pregnant women with HIV and TB infection also have a higher risk of complications such as low birth weight, preeclampsia, and preterm delivery [11,12].

In this brief research report, we would like to approach the interplay of TB and sexual and reproductive health (SRH) from the perspective of women, mostly of reproductive age, diagnosed with TB, who might experience pregnancy during treatment. We describe the contraception use at TB diagnosis and pregnancy outcomes during and after anti-TB treatment.

## 2. Materials and Methods

### 2.1. Study Design 

The data presented below are from the TB Sequel observational cohort study carried out since September 2017 in four African countries: South Africa, Tanzania, Mozambique, and The Gambia [13]. For this sub-analysis, we selected only female participants diagnosed with drug-susceptible TB. As a result, a total of 486 women were included. Data collection took place at baseline (TB diagnosis), day 14, months 2, 4 and 6 (end of treatment for drug-susceptible TB). Information was collected via questionnaires, clinical examination and laboratory tests. More details about the study design can be found in the study protocol [13]. 

### 2.2. Data Analysis

IBM SPSS Statistics version 27.0. (International Business Machines Corporation, New York, NY, USA) was used to conduct descriptive and bivariate analysis of the data. We selected socio-demographic, TB, contraception and pregnancy-related variables for the analysis. We used chi-square and Fisher’s exact test of independence to verify the existence of correlations between categorical variables with 0.05 being the threshold of significance.

## 3. Results

### 3.1. Socio-Demographic and Health Characteristics of Participants

A total of 486 women with drug-susceptible TB were recruited in the TB Sequel cohort. The majority of the participants were Black African (*n* = 478; 98.4%), born in Mozambique (*n* = 139; 28.6%) and had the age between 25 and 35 years (*n* = 199; 40.9%). The vast majority of the participants were of reproductive age (*n* = 429; 88.3%). Most of the participating women were single (*n* = 213; 43.8%). A total of 26.5% (*n* = 129) of the participants completed primary education while only 2.9% (*n* = 14) achieved an educational level of university or higher. More than half of the participants (*n* = 253/462; 54.8%) were unemployed. More than half of the participants were infected with human immunodeficiency virus (HIV) at the study baseline (*n* = 266; 54.7%), with 68.5% (*n* = 165/241) receiving antiretroviral therapy (ART) and 42.7% (*n* = 108/253) had cluster of differentiation 4 (CD4) count below 200 cells per cubic millimetre. A total of 11.7% of the participating women (*n* = 57) experienced past TB episodes. A total of 303 (62.3%) women had more than three important TB symptoms such as cough, night sweats and unintended weight loss. Most of the participating women (*n* = 416; 85.6%) were TB-cured. All socio-demographic characteristics, health status and medical history of the participating women are presented in Table 1.

### 3.2. Contraception Use

**Figure 1:**
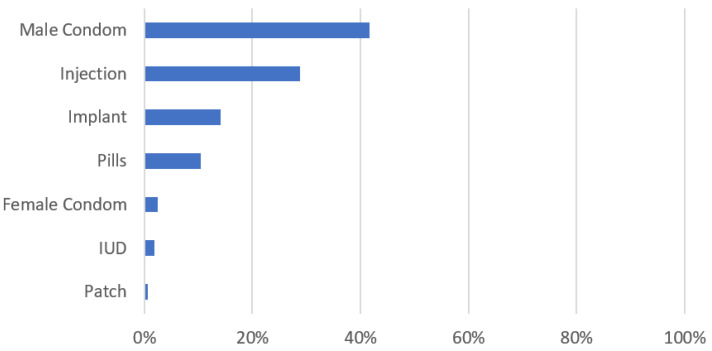
Used methods of contraception.

All participating women within the reproductive age group (18–49 years), who were not pregnant at baseline (*n* = 412/486), were asked about their contraception use. Only 40.4% (*n* = 163/403) of them used contraception. The male condom was the most preferred method of contraception (*n* = 68/163; 41.7%), while the birth control patch was the least used (*n* = 1/163; 0.6%). All methods of contraception are presented in Figure 1. Abstinence (*n* = 94/240; 39.1%), fear of the side effects (*n* = 25/240; 10.4%), wish to become pregnant (*n* = 19/240; 7.9%), menopause (*n* = 12/240; 5%) and partner disapproval (*n* = 9/240; 3.8%) were the main reasons for not using contraception.

A bivariate analysis, which excluded participants within the reproductive age group who were pregnant or experiencing menopause at baseline, indicated a correlation between contraception use and education level (*p* = 0.006). Women who completed a level above secondary school were more likely to use contraceptive methods compared to the ones who only finished secondary school. However, no significant correlation was found between contraception use on the one hand and age group (*p* = 0.262), marital status (*p* = 0.553), HIV status (*p* = 0.518) or employment (*p* = 0.927) on the other hand. There was a significant difference between countries (*p* < 0.001): with the highest reported use of contraception in women enrolled in Mozambique and lowest in The Gambia (60% and 12.5%, respectively).

### 3.3. Pregnancy during TB Treatment

#### 3.3.1. Health Status during Pregnancy

A total of 31 out of 486 participants experienced pregnancy till the end of anti-TB treatment. More than half of them were pregnant at baseline (*n* = 17/31; 54.8%), whereas one (3.2%), two (6.5%), five (16.1%) and six (19.4%) women reported pregnancy at day 14, month 2, 4, and 6 visits, respectively. The majority of them were HIV negative (*n* = 19/31; 61.3%) at baseline. Their mean haemoglobin level was 10.72 g/dL (*n* = 31) at baseline and increased to 11.75 g/dL (*n* = 25/31) till month 6 of the study. Two of them experienced past TB episodes and 61.3% did not report missing any doses of current anti-TB treatment.

#### 3.3.2. Pregnancy Outcomes

**Figure 2:**
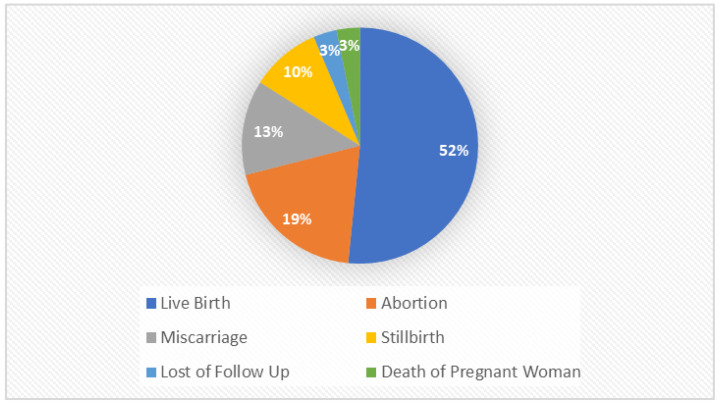
Pregnancy outcomes of the participants.

Pregnancy outcomes of 31 women varied (see Figure 2). One pregnant woman was lost to follow-up, while another participant died before the end of the study. 

When comparing the group of women who had live births (*n* = 16) to the women who had poor pregnancy outcomes (miscarriage and stillbirth, *n* = 7), it was found that women with adverse pregnancy outcomes had a higher percentage of HIV positivity and unfavourable anti-TB treatment outcomes. The relationship was not significant, but we might assume with a bigger sample size it could change. Furthermore, HIV-positive women who experienced poor pregnancy outcomes had a slightly higher percentage of CD4 count below 200 cells/mm^3^ (*n* = 2/5; 40%) compared to HIV-positive women who experienced live births (*n* = 1/3; 33.3%) with no significance (*p* = 0.714). The majority of women who had live births (87.5%) as well as poor pregnancy outcomes (71.5%) were under the age of 36 years. None of the women with poor pregnancy outcomes were underweight or smoking during pregnancy. Additionally, no significant correlation was found between age group and alcohol consumption on the one hand and pregnancy outcome on the other hand. The comparison between both groups is presented in Table 2. 

## 4. Discussion

This report addresses an important but often neglected topic of tuberculosis and sexual and reproductive health by describing findings from a TB Sequel observational cohort study in four African countries. Women, who participated in this study, were mostly of reproductive age (88.3%) and had low contraception use (40.4%). A significant number of women (31 out of 486 participants) experienced pregnancy till the end of anti-TB treatment. A relatively high percentage of them suffered from adverse pregnancy outcomes (22.5%)—miscarriage and stillbirth.

Below, we would like to draw attention to three important aspects in order to highlight the need to tackle TB and SRH, including HIV, as interlinked and complex issues. First, similar socio-economic factors often contribute to poor SRH outcomes, and higher risk of TB infection and unfavourable treatment outcomes in African countries [14,15,16,17,18,19]. The differences found in SRH services’ availability, accessibility and quality in Sub-Saharan African countries can be caused by socio-economic inequalities [19]. Low modern contraception use is also associated with lower education and income [20]. The same correlation between contraception use and education was found in our study. Furthermore, low socio-economic level increases the risk of TB infection and unsuccessful TB treatment, where mainly poverty leads to unsatisfactory TB knowledge and inadequate health seeking behaviour [21,22,23]. These factors also contribute to an increase in the probability of unsuccessful treatment outcomes, for example, in Uganda, excessive poverty and illiteracy were correlated with a failure of TB treatment [24]. Thus, addressing these social determinants, ensuring equitable access to services and information will strengthen the prevention and treatment efforts.

Second, a high percentage of TB-infected women in this cohort were also HIV positive (54.7%), with 38.7% of HIV positivity among 31 pregnant women. Taking into account a higher rate of adverse SRH outcomes in HIV and TB co-infected women described in the literature and the elevated burden of both infections in women of reproductive age, it is important to continue strengthening proper screening, diagnosis, treatment and prevention of both conditions. Moreover, as the contraception use in this cohort was relatively low, the value of offering family planning to prevent adverse pregnancy outcomes in TB infected women has to be considered for the National TB Programs [25]. Counselling women about the risks associated with becoming pregnant while undergoing TB treatment and referring them to appropriate care if they intend to conceive is essential. This should be performed by trained health professionals, as there is evidence suggesting that after being diagnosed with TB, women sometimes receive mixed and inconsistent instructions from healthcare providers regarding sexual life, pregnancy and contraception [26].

Finally, all individuals with TB frequently get stigmatized, since the infectious disease is socially linked to poor socio-economic status and unsuitable living conditions [27,28]. However, different studies show that women with TB are considerably more subjected to discrimination compared to men, which hinders their ability to receive early TB diagnosis and access effective TB treatment and services [27]. TB and HIV co-infection leads to amplified stigmatization, taking the fact that people having HIV are more likely to be infected with TB with a rate of 20 to 40 times [29]. Women suffer from that the most, especially in regions where they are unevenly infected by HIV such as Sub-Saharan African countries [30]. As another dimension of social stigma, women with TB might face a negative impact on their marriage prospects [31]. Raising awareness in the community and improving knowledge on TB-, HIV- and SRH-related issues are important steps to fight stigma.

This sub-study has limitations. Due to a small sample size of pregnant women in the cohort, we were not able to make meaningful conclusions about the contribution of TB treatment outcomes, HIV status and other relevant factors to adverse pregnancy outcomes. For future research efforts, it would be beneficial to establish a larger cohort of pregnant women with TB (and HIV co-infection) or analyse secondary data from larger TB/HIV cohorts/national registries, in order to support evidence on the effect of TB, HIV and other potential factors on SRH, especially pregnancy outcomes.

## 5. Conclusions

The aim of this research report was to raise awareness of SRH-related issues in women diagnosed with drug-susceptible TB. To conclude, addressing social and economic determinants, which are contributing to both adverse SRH outcomes and the risk of TB infection, will ensure a more efficient approach to tackling these issues. Furthermore, the linkage of SRH services with TB programs in high-burden countries and the provision of SRH guidelines and training to healthcare providers in the National TB Programs are vital to improve SRH outcomes and decrease obstetric risks during TB.

## Figures and Tables

**Table 1:** Socio-demographic characteristics, health status and medical history of the participants.

Participants Characteristics	Number (*n* = 486)	Percentage (%)
**Socio-Demographic Characteristics**		
**Age Group**		
18–24 Years	91	18.8
25–35 Years	199	40.9
36–49 Years	139	28.6
>49 Years	57	11.7
**Racial background**		
Black African	478	98.4
Mixed	7	1.4
White	1	0.2
**Country of Birth**		
Mozambique	139	28.6
Tanzania	115	23.7
The Gambia	97	20
South Africa	99	20.4
Other	36	7.4
**Marital Status**		
Single	213	43.8
Married	124	25.5
Living with Partner (Cohabitation)	57	11.7
Divorced/Separated	53	10.9
Widowed	39	8
**Educational Level**		
Never attended formal schooling	80	16.5
Primary school	129	26.5
Secondary school	124	25.5
High school (or equivalent)	127	26.1
Vocational training	12	2.5
University or higher	14	2.9
**Current Employment** (*n* = 462, 24 missing observations)		
Self-employed	53	11.5
Formally employed	58	12.5
Informally employed	22	4.8
Unemployed	253	54.8
On sick leave	45	9.7
Student at school or university	29	6.3
Retired	2	0.4
**Smoking**		
Yes	24	4.9
No	462	95.1
**Alcohol Consumption**		
Yes	121	24.9
No	365	75.1
**Health Status and Medical History**		
**BMI**		
Underweight (<18.5)	176	36.2
Normal (18.5–24.9)	242	49.8
Overweight (25.0–29.9)	45	9.3
Obese (≥30.0)	23	4.7
**HIV**		
Positive	266	54.7
Negative	220	45.3
**Anaemia** * (*n* = 479, 7 missing observations)		
Mild	87	18.2
Moderate	234	48.8
Severe	42	8.8
Normal values	116	24.2
**Past TB Episodes**		
Yes	57	11.7
No	429	88.3
**Number of important TB Symptoms**		
1	65	13.4
2	111	22.8
3+	303	62.3
No symptoms	7	1.5
**Self-reported Adherence to TB Treatment** (*n* = 344, 142 missing observations)		
Yes	305	88.7
No	39	11.3
**TB treatment outcomes**		
Cured	416	85.6
Treatment failure	26	5.3
Lost to follow-up	19	3.9
Died before the end of the study	11	2.3
Withdrawn from the study	14	2.9

* Anaemia—based on haemoglobin level in grams per litre (g/L): Mild (110–119 g/L for non-pregnant women and 100–109 g/L for pregnant women); moderate (80–109 g/L for non-pregnant women and 70–99 g/L for pregnant women); severe (<80 g/L for non-pregnant women and <70 g/L for pregnant women); normal values (≥120 g/L for non-pregnant women and ≥110 g/L for pregnant women).

**Table 2:** HIV status, TB treatment outcome, age group, BMI level, smoking status, and alcohol consumption of women who had live births vs. women who had miscarriages and stillbirths.

	Women with Live Births		Women with Miscarriages and Stillbirths		*p*-Value
	Number (*n* = 16)	Percentage (%)	Number (*n* = 7)	Percentage (%)	
**HIV Status**					0.052
HIV Positive	4	25	5	71.5	
HIV Negative	12	75	2	28.5	
**TB Treatment Outcome**					0.067
Cured	15	93.8	4	57	
Treatment failure	1	6.2	3	43	
**Age Group**					0.352
<36 years	14	87.5	5	71.5	
≥36 years	2	12.5	2	28.5	
**BMI Level**					NA
Underweight	4	25	0	0	
Not underweight	12	75	7	100	
**Smoking**					NA
Yes	2	12.5	0	0	
No	14	87.5	7	100	
**Alcohol Consumption**					0.508
Yes	4	25	1	14.3	
No	12	75	6	85.7	

## Data Availability

The dataset and materials used in this study are available from the author in correspondence on reasonable request.

## Appendix A

TB Sequel Consortium: Gavin Churchyard, Michael Hoelscher, Robert Wallis, Salome Charalambous, Kavindhran Velen, Fadzai Munedzimwe, Anna-Maria Mekota, Christof Geldmacher, Friedrich Riess, Fidelina Zekoll, Ulrich von Both, Knut Lönnroth, Stefan Niemann, Matthias Merker, Viola Dreyer, Ulrich Schaible, Christoph Leschczyk, Beate Kampmann, Jayne Sutherland, Basil Sambou, Caleb Muefong, Fatoumatta Darboe, Abi-Janet Riley, Binta Sarr, Ben Dowsing, Azeezat Sallahdeen, Shamanthi Jayasooriya, Abdou Sillah, Nyanda Elias Ntinginya, Issa Sabi, Daniel Mapamba, Mkunde Chachage, Elimina Siyame, Ian Sanne, Lyndel Singh, Jaclyn Bennet, Noluthando Mwelase, Ilesh Jani, Celso Khosa, Nilesh Bhatt, Sofia Viegas, Khalide Azam, Nadia Sitoe, Carla Madeira, Vania Maphossa, Cristovão Matusse, Pedroso Nhassengo, Denise Evans, Kamban Hirasen, Caroline Govathson, Tembeka Sineke, Sydney Rosen.
